# Supplementary material for: Impact of Season on Intestinal Bacterial Communities and Pathogenic Diversity in Two Captive Duck Species
Source: Animals (Basel). 2023 Dec 16;13(24):3879. doi: 10.3390/ani13243879 (PMC10740475; doi:10.3390/ani13243879)
Supplement: Supplementary file 1 [file animals-13-03879-s001.zip › animals-2722802-supplementary.pdf]

Table S1. The identified potentially pathogenic bacteria carried by captive Baikal teal and common teal in this study.

Pathogenic Species	Phylum	Reported Host	Symptoms
1. *Empedobacter falsenii*	Bacteroidetes	Human	Pyelonephritis, liver cirrhosis, bacteremia [72-73]
2. *Sphingomonas koreensis*	Proteobacteria	Human	Meningitis patient, peritonitis [77-78]
3. *Dietzia maris*	Actinobacteria	Avian, fish, human	Bacteremia, septic shock, prosthetic hip infection in immunocompromised patients [79-81]
4. *Campylobacter canadensis*	Epsilonbacteraeota	Whooping crane	Diarrhea [83]
5. *Arsenicicoccus dermatophilus*	Actinobacteria	Flamingo	Pododermatitis [93]
6. *Empedobacter brevis*	Bacteroidetes	Human	Malaise, nausea, vomiting [94]
7. *Flavobacterium ceti*	Bacteroidetes	Beaked whale, human	Spontaneous bacterial peritonitis [95-96]
8. *Lactococcus garvieae*	Firmicutes	Fish	Hyperacute and haemorrhagic septicemia [97]
9. *Paracoccus yeei*	Proteobacteria	Human	Peritonitis [98]
10. *Mucispirillum schaedleri*	Deferribacteres	Human, mice	Inflammation [99]

Table S2: Relative abundance of the dominant gut bacterial phyla in different groups.

Phylum	SMB	SMC	ATB	ATC	WTB	WTC	SPB	SPC	Total
**Actinobacteria**	4.74	6.17	2.57	3.50	1.56	3.57	0.63	0.20	**2.94**
**Bacteroidetes**	21.39	10.82	25.84	35.18	8.74	15.19	14.43	11.99	**18.34**
**Cyanobacteria**	0.10	0.84	1.10	0.78	1.83	4.69	2.15	1.39	**1.48**
**Epsilonbacteraeota**	0.02	0.00	0.01	0.00	0.02	0.01	0.12	0.81	**0.11**
**Firmicutes**	18.63	36.38	28.53	21.92	26.99	42.66	46.80	64.14	**34.44**
**Fusobacteria**	0.00	0.00	0.00	0.00	0.04	0.03	4.26	0.19	**0.55**
**Proteobacteria**	55.05	45.57	40.40	38.03	58.26	31.80	31.46	21.11	**41.24**
**Verrucomicrobia**	0.01	0.01	1.19	0.01	2.16	1.39	0.05	0.11	**0.61**

Table S3. Relative abundance of the dominant gut bacterial genera in different groups.

Genus	SMB	SMC	ATB	ATC	WTB	WTC	SPB	SPC	Total
Escherichia-Shigella	41.50	24.50	22.51	8.99	11.98	0.13	4.21	4.03	**16.28**
Psychrobacter	0.05	0.06	5.41	10.54	31.85	15.68	13.64	4.04	**9.29**
Sphingobacterium	12.68	7.25	13.03	19.39	1.62	4.66	2.12	0.28	**8.19**
Acinetobacter	2.36	1.60	11.35	16.24	8.36	0.48	8.88	13.28	**7.42**
Lactobacillus	1.90	1.19	2.12	8.48	0.19	0.21	3.14	6.93	**5.88**
Chryseobacterium	0.31	0.21	10.77	15.20	0.64	0.91	13.45	7.43	**5.80**
Enterococcus	1.48	5.20	5.54	0.59	4.38	3.63	6.23	11.36	**4.22**
Comamonas	4.76	10.68	1.06	1.73	0.05	0.05	3.94	0.62	**2.86**
Lysinibacillus	1.90	1.19	2.12	8.48	0.19	0.21	3.14	6.93	**2.73**
Flavobacterium	5.67	2.75	2.76	1.99	0.18	3.67	1.52	2.72	**2.62**
Ralstonia	0.00	0.00	0.00	0.00	7.89	10.52	0.33	3.33	**2.25**
Clostridium sensu stricto 1	0.41	0.46	0.61	0.36	1.33	0.13	8.36	9.84	**2.05**
Bacteroides	0.21	0.16	0.23	0.00	5.11	5.06	1.65	6.51	**1.87**
Exiguobacterium	0.08	0.10	2.66	3.33	1.49	1.08	2.06	1.51	**1.48**
Sporosarcina	2.39	8.08	0.10	0.00	0.48	0.00	0.00	0.00	**1.42**
Empedobacter	5.72	1.79	1.05	0.12	0.00	0.00	0.00	0.00	**1.27**
Chryseomicrobium	0.00	0.01	5.94	1.48	0.00	0.00	0.00	0.00	**1.15**
Paenibacillus	2.26	0.41	0.65	2.33	0.95	0.18	0.79	1.74	**1.14**
Gottschalkia	0.05	0.07	4.25	0.07	0.61	0.22	1.44	1.47	**1.07**

Table S4. NMDS and ANOSIM test of difference groups.

Group	Stress	ANOSIM: R	ANOSIM: P value
**SMB vs SMC**	0.0586	0.120	0.1582
**ATB vs ATC**	0.0477	0.157	0.1603
**WTB vs WTC**	0.2756	0.009	0.1681
**SPB vs SPC**	0.1411	0.020	0.1663
**SMB vs ATB**	0.1551	0.4875	0.001
**SMB vs WTB**	0.1427	0.7013	0.001
**SMB vs SPB**	0.0815	0.9297	0.001
**ATB vs WTB**	0.1427	0.7013	0.001
**ATB vs SPB**	0.1131	0.8592	0.001
**WTB vs SPB**	0.1684	0.3028	0.001
**SMC vs ATC**	0.1096	0.5532	0.001
**SMC vs WTC**	0.0831	0.7306	0.001
**SMC vs SPC**	0.0963	0.7287	0.001
**ATC vs WTC**	0.0827	0.9566	0.001
**ATC vs SPC**	0.1014	0.6900	0.001
**WTC vs SPC**	0.1488	0.6265	0.001

Table S5. Co-occurrence network topological features statistics.

	SMB	SMC	ATB	ATC	WTB	WTC	SPB	SPC
**Nodes**	829	945	925	525	1,061	1,252	952	961
**Edges**	31,944	40,447	41,290	15,842	70,271	72,826	41,618	58,906
**Density**	0.093	0.091	0.097	0.115	0.125	0.093	0.092	0.128
**Diameter**	5	5	5	6	5	5	5	6
**Modularity**	1.360	1.351	1.248	1.027	0.917	1.784	1.645	1.028
**Average degree**	77.066	85.602	89.276	60.350	132.462	116.335	87.433	122.593
**Average weighted**	55.349	56.948	70.592	54.044	144.117	52.291	49.908	107.209
**Average clustering coefficient**	0.806	0.781	0.825	0.833	0.856	0.766	0.779	0.833
**Average Path length**	2.633	2.575	2.666	2.750	2.448	2.594	2.561	2.523
**Number of community**	16	18	15	29	15	12	16	15

Table S6. Classification information of keystone taxa in gut bacterial co-occurrence network.

Groups	OTU	Zi	Pi	Phylum	Genus	Species
**SMB**	OTU960	-5.740	0.813	Actinobacteria	*Nocardioides*	Unclassified
	OTU744	-2.374	0.760	Firmicutes	*Staphylococcus*	Unclassified
	OTU902	-2.883	0.724	Proteobacteria	*Pseudomonas*	Unclassified
	OTU684	-2.337	0.715	Firmicutes	*Peptococcus*	Unclassified
	OTU664	-2.883	0.704	Chloroflexi	Unclassified	Unclassified
	OTU230	-4.297	0.704	Firmicutes	*Macrococcus*	Unclassified
	OTU558	-1.935	0.697	Bacteroidetes	*Sphingobacterium*	Unclassified
	OTU87	-2.345	0.672	Firmicutes	*Gottschalkia*	Unclassified
	OTU552	-4.728	0.671	Bacteroidetes	*Sphingobacterium*	Unclassified
	OTU1162	-2.745	0.653	Proteobacteria	*Pseudomonas*	Unclassified
	OTU93	3.876	0.1439	Proteobacteria	Unclassified	Unclassified
**SMC**	OTU32	-2.941	0.797	Firmicutes	*Trichococcus*	Unclassified
	OTU133	-2.998	0.750	Proteobacteria	*Devosia*	Unclassified
	OTU684	-2.769	0.729	Firmicutes	*Peptococcus*	Unclassified
	OTU3213	-2.884	0.714	Proteobacteria	*Paracoccus*	Unclassified
	OTU41	-2.176	0.710	Firmicutes	*Psychrobacillus*	Unclassified
	OTU87	-2.197	0.689	Firmicutes	*Gottschalkia*	Unclassified
	OTU1105	-2.076	0.676	Firmicutes	*Lachnoclostridium*	Unclassified
	OTU53	-2.176	0.669	Firmicutes	*Psychrobacillus*	Unclassified
	OTU707	-2.609	0.648	Bacteroidetes	*Flavobacterium*	*Flavobacterium sp.*
	OTU180	-2.142	0.646	Firmicutes	*Paenibacillus*	*Paenibacillus xylanilyticus*
	OTU1130	-2.826	0.643	Proteobacteria	*Allorhizobium-Neorhizobium-Pararhizobium-Rhizobium*	Unclassified
	OTU2202	-2.747	0.640	Bacteroidetes	*Chryseobacterium*	Unclassified
	OTU596	-7.158	0.633	Proteobacteria	Unclassified	Unclassified
	OTU1090	-1.482	0.626	Firmicutes	Unclassified	Unclassified
**ATB**	OTU280	-2.922	0.704	Firmicutes	*Paenibacillus*	Unclassified
	OTU848	-2.820	0.689	Firmicutes	*Lactobacillus*	Unclassified
	OTU156	-3.073	0.689	Bacteroidetes	*Parasegetibacter*	Unclassified
	OTU155	-2.922	0.679	Chloroflexi	*Paraburkholderia tropica*	*Paraburkholderia tropica*

Groups	OTU	Zi	Pi	Phylum	Genus	Species
	OTU558	3.647	0.0453	Bacteroidetes	*Sphingobacterium*	Unclassified
	OTU560	3.770	0.0228	Firmicutes	*Lysinibacillus*	Unclassified
	OTU566	3.770	0.0228	Proteobacteria	*Stenotrophomonas*	*Stenotrophomonas koreensis*
	OTU569	3.893	0.0226	Bacteroidetes	*Flavobacterium*	Unclassified
	OTU1813	4.07656229	0.0222	Bacteroidetes	*Chryseobacterium*	Unclassified
	OTU732	4.077	0.0222	Proteobacteria	*Noviherbaspirillum*	Unclassified
	OTU797	4.077	0.0222	Bacteroidetes	*Pedobacter*	Unclassified
	OTU571	4.138	0.0221	Bacteroidetes	*Sphingobacterium*	Sphingobacterium sp.
	OTU559	4.199	0.0219	Proteobacteria	*Brevundimonas*	Unclassified
**ATC**	OTU1773	-1.818	0.688	Bacteroidetes	*Chryseobacterium*	Unclassified
	OTU106	-1.861	0.633	Actinobacteria	*Oerskovia*	Unclassified

**WTB**	OTU284	-1.825	0.813	Firmicutes	*Erysipelotrichaceae UCG-003*	Unclassified
	OTU412	-3.515	0.796	Firmicutes	*Peptococcus*	Unclassified
	OTU1449	-3.302	0.734	Verrucomicrobia	*Akkermansia*	Unclassified
	OTU126	-3.159	0.728	Proteobacteria	*Massilia*	Unclassified
	OTU1414	-2.475	0.687	Actinobacteria	*Curtobacterium*	Unclassified
	OTU2746	-1.760	0.655	Proteobacteria	*Pseudomonas*	Unclassified
	OTU27	-1.804	0.653	Proteobacteria	*Comamonas*	Unclassified
	OTU56	-2.442	0.645	Proteobacteria	*Porphyrobacter*	Unclassified
**WTC**	OTU23	-2.802	0.778	Firmicutes	*Enterococcus*	Unclassified
	OTU2325	-2.698	0.690	Cyanobacteria	Unclassified	Unclassified
	OTU34	-2.592	0.679	Firmicutes	*Lactobacillus*	*Lactobacillus aviarius*
	OTU1276	-2.592	0.670	Firmicutes	*Parvimonas*	Unclassified
	OTU1955	-2.910	0.670	Epsilonbacteraeota	*Arcobacter*	Unclassified
	OTU31	-2.521	0.652	Firmicutes	*Lactobacillus*	*Lactobacillus aviarius*
	OTU69	-2.345	0.646	Proteobacteria	*Sphingobium*	Unclassified
	OTU360	-2.707	0.639	Firmicutes	*Enterococcus*	*Enterococcus columbae*
	OTU247	-2.381	0.638	Proteobacteria	Unclassified	Unclassified
	OTU106	-2.744	0.628	Actinobacteria	*Oerskovia*	Unclassified
	OTU626	-2.662	0.624	Firmicutes	Unclassified	Unclassified

Groups	OTU	Zi	Pi	Phylum	Genus	Species
	OTU862	3.228	0.077	Actinobacteria	Varibaculum	Unclassified
	OTU155	3.160	0.106	Chloroflexi	*Paraburkholderia*	*Paraburkholderia tropica*
	OTU205	3.160	0.106	Proteobacteria	*Methyloparacoccus*	Unclassified
**SPB**	OTU40	-4.569	0.801	Firmicutes	*Paenibacillus*	Unclassified
	OTU187	-2.304	0.778	Bacteroidetes	*Dyadobacter*	Unclassified
	OTU538	-2.712	0.760	Cyanobacteria	*Chroococcidiopsis PCC 7203*	Unclassified
	OTU67	-3.178	0.754	Proteobacteria	*Ralstonia*	Unclassified
	OTU75	-2.429	0.733	Proteobacteria	*Escherichia-Shigella*	Unclassified
	OTU181	-2.382	0.717	Bacteroidetes	*Parabacteroides*	Unclassified
	OTU366	-2.241	0.716	Proteobacteria	*Pseudomonas*	Unclassified
	OTU495	-2.523	0.704	Bacteroidetes	*Prevotellaceae Ga6A1 group*	Unclassified
	OTU277	-2.178	0.689	Firmicutes	*Subdoligranulum*	Unclassified
	OTU2381	-1.831	0.684	Proteobacteria	*Duganella*	Unclassified
	OTU412	-2.272	0.679	Firmicutes	*Peptococcus*	Unclassified
	OTU1648	-2.240	0.671	Firmicutes	*Gottschalkia*	Unclassified
	OTU64	-2.272	0.668	Proteobacteria	*Ralstonia*	Unclassified
	OTU962	-1.989	0.661	Firmicutes	Unclassified	Unclassified
	OTU217	-2.571	0.633	Cyanobacteria	Unclassified	Unclassified
**SPC**	OTU411	-1.713	0.730	Proteobacteria	*Massilia*	Unclassified
	OTU684	-1.713	0.728	Firmicutes	*Peptococcus*	Unclassified
	OTU2661	-1.713	0.686	Proteobacteria	*FukuN57*	Unclassified
	OTU3027	-1.735	0.680	Firmicutes	*Ruminococcaceae UCG-014*	Unclassified
	OTU2459	-1.735	0.672	Firmicutes	*Blautia*	bacterium ic1272
	OTU1517	-2.016	0.640	Firmicutes	Unclassified	Unclassified

OTU297	-1.649	0.636	Proteobacteria	*Allorhizobium-Neorhizobium-Pararhizobium-Rhizobium*	Unclassified
OTU2189	-1.454	0.631	Proteobacteria	*Bradyrhizobium*	Unclassified

Table S7. The intestinal bacterial and potentially pathogenic sequences across the samples.

Sample ID	Sequences		Pathogen/Bacteria (%)
	Bacteria	Pathogen	
SMB1	32,968.00	821	2.49
SMB2	32,373.00	1259	3.89
SMB3	32,042.00	1526	4.76
SMB4	55,459.00	75	0.14
SMB5	47,630.00	1703	3.58
SMB6	40,289.00	138	0.34
SMB7	36,193.00	1333	3.68
SMB8	39,080.00	21	0.05
SMB9	35,555.00	3521	9.90
SMB10	55,249.00	28	0.05
SMB11	42,649.00	19	0.04
SMB12	36,144.00	138	0.38
SMC1	35,053.00	35	0.10
SMC2	28,615.00	30	0.10
SMC3	51,637.00	34	0.07
SMC4	45,631.00	137	0.30
SMC5	29,241.00	348	1.19
SMC6	38,612.00	1069	2.77
SMC7	45,568.00	300	0.66
SMC8	28,974.00	437	1.51
SMC9	42,384.00	522	1.23
SMC10	47,678.00	759	1.59
SMC11	47,285.00	339	0.72
SMC12	31,962.00	276	0.86
ATB1	40,702.00	50	0.12
ATB2	14,955.00	27	0.18
ATB3	21,121.00	32	0.15
ATB4	17,014.00	53	0.31
ATB5	20,525.00	42	0.20
ATB6	12,850.00	147	1.14
ATB7	18,655.00	39	0.21
ATB8	27,297.00	51	0.19
ATB9	20,762.00	12	0.06
ATB10	17,865.00	0	0.00
ATB11	19,801.00	2	0.01
ATB12	22,781.00	30	0.13

Sample ID	Sequences		Pathogen/Bacteria (%)
	Bacteria	Pathogen	
ATC1	17,446.00	4	0.02
ATC2	18,078.00	0	0.00
ATC3	27,494.00	2	0.01
ATC4	22,735.00	4	0.02
ATC5	17,936.00	2	0.01
ATC6	18,781.00	2	0.01
ATC7	20,931.00	3	0.01
ATC8	13,769.00	22	0.16
ATC9	22,317.00	86	0.39
ATC10	15,234.00	0	0.00
ATC11	20,430.00	9	0.04
ATC12	14,712.00	8	0.05
WTB1	41,301.00	0	0.00
WTB2	30,533.00	2	0.01
WTB3	57,421.00	1	0.00
WTB4	29,319.00	0	0.00
WTB5	50,024.00	6	0.01
WTB6	54,528.00	0	0.00
WTB7	45,980.00	0	0.00
WTB8	26,398.00	9	0.03
WTB9	38,712.00	5	0.01
WTB10	16,101.00	0	0.00
WTB11	47,295.00	0	0.00
WTB12	38,809.00	2	0.01
WTC1	44,822.00	2	0.00
WTC2	52,193.00	28	0.05
WTC3	53,108.00	0	0.00
WTC4	37,336.00	8	0.02
WTC5	24,502.00	1	0.00
WTC6	45,874.00	5	0.01
WTC7	40,057.00	0	0.00
WTC8	58,421.00	0	0.00
WTC9	33,103.00	3	0.01
WTC10	55,085.00	4	0.01
WTC11	39,439.00	5	0.01
WTC12	46,355.00	0	0.00

Sample ID	Sequences		Pathogen/Bacteria (%)
	Bacteria	Pathogen	
SPB1	35,722.00	20	0.06
SPB2	53,840.00	0	0.00
SPB3	30,262.00	2	0.01
SPB4	19,281.00	20	0.10
SPB5	30,608.00	17	0.06
SPB6	32,659.00	0	0.00
SPB7	47,027.00	2	0.00
SPB8	59,335.00	3	0.01
SPB9	39,525.00	1	0.00
SPB10	50,575.00	0	0.00
SPB11	49,452.00	1	0.00
SPB12	27,484.00	3	0.01
SPC1	51,267.00	39	0.08
SPC2	36,617.00	4	0.01
SPC3	43,456.00	0	0.00
SPC4	45,010.00	5	0.01
SPC5	37,223.00	1	0.00
SPC6	30,820.00	0	0.00
SPC7	37,578.00	48	0.13
SPC8	63,959.00	2	0.00
SPC9	49,965.00	1	0.00
SPC10	31,577.00	0	0.00
SPC11	27,781.00	0	0.00
SPC12	23,473.00	8	0.03

Table S8. The difference in the relative abundance of potentially pathogenic bacteria observed among different groups.

Pathogenic species	SMB	SMC	ATB	ATC	WTB	WTC	SPB	SPC
Empedobacter falsenii	5.7630 (7.8323)[b]	1.5091 (1.4692)[a]	0.2617 (0.3012)[a]	0.0658 (0.1720)[a]	0.0000 (0.0000)[a]	0.0000 (0.0000)[a]	0.0000 (0.0000)[a]	0.0000 (0.0000)[a]
Arsenicicoccus dermatophilus	0.0000 (0.0000)[a]	0.0013 (0.0030)[b]	0.0000 (0.0000)[a]	0.0000 (0.0000)[a]	0.0000 (0.0000)[a]	0.0000 (0.0000)[a]	0.0000 (0.0000)[a]	0.0000 (0.0000)[a]
Campylobacter canadensis	0.0052 (0.0117)[a]	0.0020 (0.0049)[a]	0.0000 (0.0000)[a]	0.0013 (0.0045)[a]	0.0104 (0.0201)[a]	0.0098 (0.0111)[a]	0.0313 (0.0000)[ab]	0.0690 (0.1288)[b]
Dietzia maris	0.0020 (0.0049)[a]	0.0117 (0.0312) [a]	0.0332 (0.0622)[a]	0.0221 (0.0406) [a]	0.0046 (0.0188) [a]	0.0260 (0.0596) [a]	0.0026 (0.0069) [a]	0.0000 (0.000) [a]
Empedobacter brevis	0.0033 (0.0113) [a]	0.0000 (0.0000) [a]	0.0000 (0.0000) [a]	0.0000 (0.0000) [a]	0.0000 (0.0000) [a]	0.0000 (0.0000) [a]	0.0000 (0.0000) [a]	0.0000 (0.0000) [a]
Flavobacterium ceti	0.4154 (0.8257) [b]	0.0391 (0.1165) [a]	0.0000 (0.0000) [a]	0.0000 (0.0000) [a]	0.0000 (0.0000) [a]	0.0000 (0.0000) [a]	0.0000 (0.0000) [a]	0.0000 (0.0000) [a]
Lactococcus garvieae	0.0013 (0.0045) [a]	0.0007 (0.0023) [a]	0.0000 (0.0000) [a]	0.0000 (0.0000) [a]	0.0000 (0.0000) [a]	0.0007 (0.0023) [a]	0.0000 (0.0000) [a]	0.0000 (0.0000) [a]
Stenotrophomonas koreensis	0.6973 (1.0577) [ab]	1.2240 (2.1499) [b]	0.0202 (0.0289) [a]	0.0026 (0.0090) [a]	0.0000 (0.0000) [a]	0.0000 (0.0000) [a]	0.0104 (0.0361) [a]	0.0007 (0.0023) [a]
Paracoccus yeei	0.0000 (0.0000) [a]	0.0026 (0.0051) [b]	0.0007 (0.0023) [a]	0.0007 (0.0023) [a]	0.0000 (0.0000) [a]	0.0000 (0.0000) [a]	0.0000 (0.0000) [a]	0.0000 (0.0000) [a]
Mucispirillum schaedleri	0.0020 (0.0049) [a]	0.0000 (0.0000) [a]	0.0000 (0.0000) [a]	0.0000 (0.0000) [a]	0.0013 (0.0045) [a]	0.0000 (0.0000) [a]	0.0007 (0.0023) [a]	0.0007 (0.0023) [a]

The values in brackets represent the standard deviation. Letters following brackets represent significant differences from the one-way ANOVA and Duncan test ($p < 0.05$). SMB (summer Baikal teal), SMC (summer common teal), ATB (autumn Baikal teal), ATC (autumn common teal), WTB (winter Baikal teal), WTC (winter common teal), SPB (spring Baikal teal), and SPC (spring common teal).

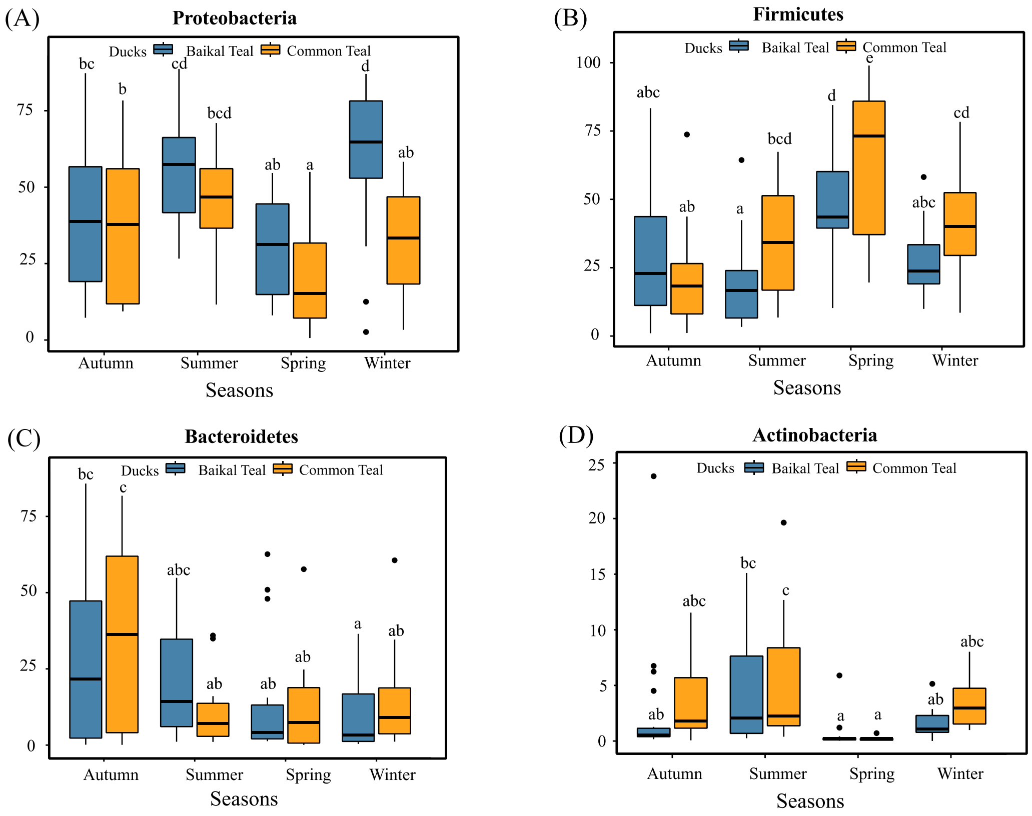

Figure S1. One-way ANOVA with Duncan's post-hoc test showed significant differences in relative abundance across groups at the phyla level. Small letters were used to indicate significant differences at $p < 0.05$. The whiskers extended from the box to the minimum and maximum values and the black dots showed the extreme values.

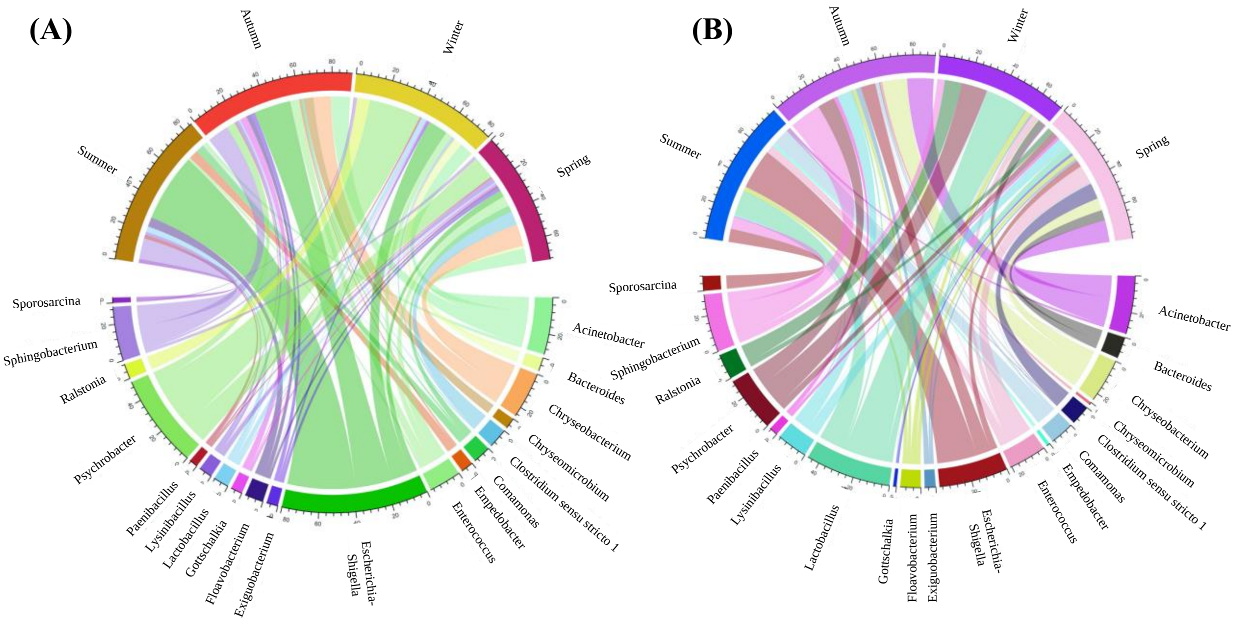

Figure S2. The chord diagram illustrates the proportionate distributions of the dominant bacterial genera in two captive ducks during four seasons. (**A**) Dominant genera in Baikal teal and (**B**) dominant genera in common teal.
